# Age and vocabulary knowledge differentially influence the N400 and theta responses during semantic retrieval

**DOI:** 10.1016/j.dcn.2023.101251

**Published:** 2023-05-02

**Authors:** Julie M. Schneider, Sonali Poudel, Alyson D. Abel, Mandy J. Maguire

**Affiliations:** a Louisiana State University, USA; b The University of Texas at Austin, USA; c San Diego State University, USA; d The University of Texas at Dallas, USA

**Keywords:** EEG, Semantic retrieval, N400, Theta, Language development

## Abstract

Using electroencephalography (EEG) to study the neural oscillations supporting language development is increasingly common; however, a clear understanding of the relationship between neural oscillations and traditional Event Related Potentials (ERPs) is needed to disentangle how maturation of language-related neural networks supports semantic processing throughout grade school. Theta and the N400 are both thought to index semantic retrieval but, in adults, are only weakly correlated with one another indicating they may measure somewhat unique aspects of retrieval. Here, we studied the relationship between the N400 amplitude and theta power during semantic retrieval with key indicators of language abilities including age, vocabulary, reading comprehension and phonological memory in 226 children ages 8–15 years. The N400 and theta responses were positively correlated over posterior areas, but negatively correlated over frontal areas. When controlling for the N400 amplitude, the amplitude of the theta response was predicted by age, but not by language measures. On the other hand, when controlling theta amplitude, the amplitude of the N400 was predicted by both vocabulary knowledge and age. These findings indicate that while there is a clear relationship between the N400 and theta responses, they may each index unique aspects of development related to semantic retrieval.

## 1 Introduction

For decades, the N400 has been the gold standard EEG measure of semantic processing (for review, see Kutas, 2011); however, in recent years, there has been a growing interest in using time-frequency analyses to investigate the theta band (typically 4–8 Hz) in relation to semantic processing and semantic development. This interest is partly driven by the capability of time-frequency analyses to identify synchronous, yet independent, neural processes supporting semantic development beyond those identified by traditional ERP measures. Preliminary evidence suggests that, although the N400 and theta are evoked by similar semantic tasks, they may be measuring unique aspects of semantic processing (Braunstein et al., 2012, Mellem et al., 2013, Schneider et al., 2016, Schneider et al., 2018, Schneider and Maguire, 2018a, Schneider and Maguire, 2019). Recent findings also suggest that compared to the N400, theta might be more sensitive to developmental differences in the neural underpinnings of semantic processing (Schneider and Maguire, 2018b). Despite these findings, studies investigating developmental changes in both the N400 and theta during semantic retrieval are sparse. The current study addresses these issues by investigating the relationship between the N400 and theta during semantic retrieval, while also considering how various developmental factors, such as language abilities and age, may modulate this relationship.

There is a longstanding history supporting the relationship between the N400 and semantic processing (for review, see Kutas and Federmeier, 2011). Studies of semantic processing in both adults and children report increased N400 amplitudes (more negative) in response to contextually incongruent compared to congruent sentence final words (Kutas and Hillyard, 1980), semantic violations in discourse (Kutas and Federmeier, 2011), and in semantically incongruent versus congruent word pairs (Chen et al., 2014, Maguire et al., 2010). From these studies, the amplitude of the N400 has been interpreted as reflecting the relative difficulty of contextual integration or retrieving semantic information from working memory (Sivonen et al., 2006). The N400 effect is seen as early as 12–14 months of age (Friedrich and Friederici, 2004) and has been reported across multiple age groups (Chen et al., 2014, Hahne et al., 2004, Henderson et al., 2011, Holcomb et al., 1992, Torkildsen et al., 2007). This suggests that the neural architecture supporting the N400 mechanism is present quite early in development and reflects semantic processing across the course of development.

While the N400 effect is a well-established measure of semantic processing, there are indications that it might not capture all of the neural response that a semantic processing task engages. Additional insights about the neural underpinnings of semantic processing and semantic development may be revealed by examining changes in the theta frequency of the EEG. Specifically, time frequency analysis of the EEG may contain additional neural information that is independent of ERPs (Cohen, 2014, Davidson and Indefrey, 2007, Schneider and Maguire, 2018a). ERPs, due to the process of averaging, only constitute neural signal which is phase-aligned to the event of interest. Time-frequency analyses, on the other hand, produce an event related measure of spectral power (frequency power changes) that reflects both phase-aligned and non-phase aligned oscillatory activities. Since time-frequency analysis decomposes the EEG signal into discrete frequencies, it also allows for the examination of overlapping neural processes (e.g., firing of neural assemblies in both theta and beta during single word processing). As such, studying frequency power changes along with ERPs has the potential to reveal additional and somewhat complementary insights about the neural underpinnings of language tasks (Bastiaansen and Hagoort, 2015, Cohen, 2014, Davidson and Indefrey, 2007, Maguire and Abel, 2013, Wang, 2010).

In the adult literature, there has been an effort to explicitly study the relationship between theta (4–8 Hz) and the N400 (Davidson and Indefrey, 2007, Schneider and Maguire, 2018a). While these studies highlight similarities between the N400 and theta engagement during semantic processing they also suggest differences in what both neural indices index during semantic processing. EEG studies report that both the N400 and theta (4–8 Hz) amplitudes increase in response to semantically incongruent relative to semantically congruent stimuli in the sentence-level (Bastiaansen et al., 2012, Bastiaansen and Hagoort, 2006, Bastiaansen and Hagoort, 2015, Davidson and Indefrey, 2007, Hagoort et al., 2004, Hald et al., 2006, Schneider et al., 2016, Schneider and Maguire, 2019) as well as at the word-level (Dini et al., 2022, Maguire et al., 2010, Roehm et al., 2004). Similarly, findings from MEG studies also report similar patterns of N400 and theta engagement during adult semantic processing (Kielar et al., 2014, Wang et al., 2012). These findings suggest that the N400 and theta may reflect similar underlying neural processes.

Although there are similarities between the N400 and theta responses, there is evidence that the N400 and the theta response are not simply the same signal analyzed in different ways, In fact, they may be the result of somewhat different neural underpinnings. For instance, studies examining both the N400 and theta responses during semantic tasks report distinct scalp topographies, corroborating that the N400 and theta components measure distinct aspects of neural activity that may arise in distinct cortical populations (Bastiaansen and Hagoort, 2015, Davidson and Indefrey, 2007, Hald et al., 2006, Kielar et al., 2014). Additionally, some studies have reported changes in the N400 without corresponding changes in the theta response (Mellem et al., 2013), while others indicate two distinct theta effects underlying the N400 effect (Roehm et al., 2004). In directly investigating the relationship between theta and the N400 in response to sentences with and without semantic violations, Schneider and Maguire (2018a) found that theta power changes could explain 25% of the variance in the N400 amplitude difference between conditions. The authors interpreted this relationship to mean that oscillatory dynamics may include neural processes beyond those identified by ERP components. Complementary to this finding, Roehm and colleagues (2004) argue that, beyond the N400-type changes related to semantic error identification, changes in theta index additional neural processes that support semantic retrieval or another cognitive process necessary to complete the task. Taken together, existing findings indicate that even if the N400 and changes in theta occur in response to the same types of semantic tasks or stimuli, they index somewhat distinct aspects of neural processes underlying semantic processing. Here we examine similarities and differences in how the N400 and theta are engaged during semantic retrieval in school-aged children (8–16 years) as a means of providing information about the ongoing development of the neural underpinnings supporting semantic processing, and their relation to other developmental factors, namely language ability and age.

Studies utilizing both ERPs and frequency power changes related to semantic development in children are relatively scarce. The limited research suggests that the N400 and theta might develop along different trajectories. Schneider and Maguire (2018b) compared the N400 and theta responses to sentences with and without semantic errors across three age groups: 8–9-years-old, 12–13-years-old children and adults. When comparing the neural response for sentences with semantic errors versus semantically correct sentences between age groups (8–9 years old vs 12–13 years old), there were no significant differences in the N400 effect but increases in theta power became more robust as a factor of age. This suggests that changes in theta frequency might be sensitive to age-related differences in the neural underpinnings of semantic processing that the N400 is not. Similarly, Schneider et al. (2016) found both the N400 and theta responses to syntactic errors in 10–12-year-old children; however, the amplitudes of these responses were not associated. While these studies have pointed to variations in how language skills and age influence the development of language-related neural networks, it remains unknown how each of these factors assert their influence and relate to behavioral outcomes. Addressing this gap can shed light onto the maturation of language-related neural networks and how they impact semantic processing throughout grade school.

The goal of the current study is to examine both the N400 and theta response with respect to semantic retrieval in school-aged children, specifically, the retrieval of individual word meanings while reading a sentence presented word-by-word. Toward this end, we focus on processing of open-class, semantically rich words (e.g., nouns, verbs, and adjectives) which reliably elicit an N400 (Münte et al., 2001) and a theta response (Bastiaansen et al., 2005). Based on our interest in examining individual differences related to age and language abilities across children, we used single-trial multilevel modeling to model the influence of these variables on measures of neural activity (N400, Theta) during semantic retrieval.

## 2 Methods

### 2.1 Participants

Two-hundred and twenty-six children, ages 8–15 years (*M*age = 11.31, *SD*age = 2.23, Females = 119), participated in the current study. All children were right-handed and free from history of traumatic brain injury, other significant neurological disorders (CVA, seizure disorders, history of high fevers, tumors), and/or language or learning disorders. While children’s linguistic experiences varied, all children were highly proficient English speakers, enrolled in English-speaking classrooms. Information related to maternal education was collected via the guardian’s self-report, and sorted into seven categories: less than 7th grade, less than 9th grade, partial high school, high school graduate (both GED and diploma), partial college/Associate’s degree, college graduate/Bachelor’s degree, graduate degree. Guardians also self-reported their children’s race (American Indian or Alaskan Native (*N* = 2); Asian (*N* = 16); Black (*N* = 27); White (*N* = 179); Multiple (*N* = 44); Other/Did not declare (*N* = 2)) and ethnicity (Hispanic or Latino (*N* = 136); Not Hispanic or Latino (*N* = 137); Did not declare (*N* = 1)). Details of participant demographic information are provided in Table 1.

**Table 1:** Demographic information stratified by age (categorical). One-way ANOVAs conducted on each variable of interest indicate that age groups only differed in chronological age in year (continuous), but not in their performance on behavioral assessments or in their maternal education. All variables were either centered at their mean or z-scored in subsequent analyses.

	**8–9**	**10–11**	**12–13**	**14–15**	**p-value**
***N***	76	66	43	41	
**Chronological Age in years** (mean (SD))	8.88 (0.57)	10.93 (0.52)	12.92 (0.57)	14.73 (0.58)	< 0.001
**Vocabulary: PPVT standard score** (mean (SD))	108.72 (18.22)	110.32 (19.41)	107.44 (16.63)	103.17 (16.24)	0.241
**Reading Comprehension: GORT scaled score)** (mean (SD))	9.69 (3.00)	10.23 (3.64)	10.20 (3.72)	9.10 (3.14)	0.327
**Phonological Memory: Non-word repetition % Consonants Correct** (mean (SD))	89.08 (10.65)	89.92 (11.05)	89.69 (9.51)	91.32 (5.59)	0.707
**Ethnicity**					0.484
Not Hispanic/Latino	48	38	25	26	
Hispanic/Latino	48	43	25	20	
**Language Experience** (*N* bilingual (percent))	42 (55.3)	39 (59.1)	20 (46.5)	18 (43.9)	0.360
**Maternal Education**					0.864
< 7th Grade	2	3	3	2	
< 9th Grade	6	4	5	2	
Partial HS	3	3	2	3	
HS Graduate	8	6	7	8	
Partial College	16	9	4	6	
College Graduate	24	26	16	11	
Graduate Degree	17	15	6	9	
**Race**					0.982
American Indian or Alaskan Native	1	0	1	0	
Asian	5	6	3	2	
Black	11	7	4	5	
White	60	52	34	33	
Multiple	17	13	9	5	

Parental consent and child assent was obtained in accordance with the Institutional Review Board at the University of Texas at Dallas. This study was conducted according to the Good Clinical Practice Guidelines, the Declaration of Helsinki, and the U.S. Code of Federal Regulations.

### 2.2 Measures of language ability

Children completed behavioral assessments that provided measures of receptive vocabulary, reading comprehension, and phonological memory. The Peabody Picture Vocabulary Test– Fourth Edition (PPVT-4;Dunn and Dunn, 1997) was used to measure receptive vocabulary. Reading comprehension was measured using the Gray Oral Reading Tests-Fifth Edition reading comprehension subtest (GORT-5; Wiederholt and Bryant, 2012). Considering the simple view of reading, in which comprehension is predicted by fluency, accuracy, and rate (Hoover and Gough, 1990) and is critical to subsequent vocabulary development in grade school (Cain et al., 2004), in the current study we focused only on the reading comprehension subtest of the GORT-5, which requires participants to read passages aloud and answer related questions. Phonological memory was measured using the Nonword Repetition task (Dollaghan and Campbell, 1998). For the PPVT-4 and GORT-5, available standard/scaled scores were used. For the NWR, percentage of correctly produced consonants was used. In subsequent analyses, all assessment scores were z normed. Average scores on each measure stratified across age groups are included in Table 1.

### 2.3 EEG Task

#### 2.3.1 Stimuli

The current study investigates the neural response to content words introduced in sentence triplets, which come from a larger word inferencing task used in previous studies (Abel et al., 2018, Maguire et al., 2018, Ralph et al., 2020, Schneider et al., 2022). In this study, children read 100 sets of sentence triplets presented word-by-word on a computer screen. Sentences were between 6 and 9 words in length. To ensure task performance was not attributed to differences in children’s existing knowledge of the words in each sentence, we only included early acquired, high frequency words from well-established corpora (Carroll et al., 1971, Fenson et al., 1994). Individual content words, or words carrying meaning (nouns, main verbs, adjectives and adverbs), were extracted from each sentence and included in the current analysis (for example, see Table 2). For information on how the stimuli were created see (Abel et al., 2018).

**Table 2:** Content words embedded in sentence triplet. *Note:* The nonword “*bown*” represents the real word “oven”. Words highlighted in red represent content words which were included in the current analysis.

Meaning Acquisition Task	
Sentence 1	It gets hot when we turn on the *bown*.
Sentence 2	Dinner is cooking in the *bown*.
Sentence 3	She baked the cookies in the *bown*.

#### 2.3.2 Procedure

Participants sat in a chair 1 m from a computer monitor. They were told that they would read sets of three sentences presented word-by-word with a made-up target word as the last word in each sentence. Each word in the sentence appeared for 500 ms. A blank screen appeared between words for 300 ms, except for the blank screen directly preceding the target word, which was presented for 600 ms. The current analysis is interested in neural engagement during processing of content words presented in a sentence context. Therefore, only real, content words presented in each sentence are included in the analysis.

### 2.4 EEG acquisition

Continuous EEG data was collected from 64 silver/silver-chloride electrodes mounted within an elastic cap (Neuroscan Quickcap) configured in accordance with the International 10–20 electrode placement standard (Compumedics, Inc.). EEG data were recorded continuously using a Neuroscan SynAmps2 amplifier and CURRY Neuroimaging software, sampled at 1 kHz with impedances typically below 5 kΩ. Data were recorded with the ground at Fz and the reference electrode located near the vertex.

### 2.5 EEG pre-processing

Continuous EEG was collected at a rate of 1000 Hz with Neuroscan Neuroimaging Suite software and analyzed within the EEGlab toolbox of Matlab (Delorme and Makeig, 2004). All continuous data was high-pass filtered offline at 0.1 Hz, low-pass filtered at 50 Hz, and resampled down to 500 Hz. Bad electrodes were spherically interpolated and areas of muscle activity/artifacts were removed from the continuous EEG data by using the clean raw data plug-in within EEGlab. Subsequently, data was decomposed using an Independent Components Analysis (ICA; Delorme et al., 2001). ICA helps ensure that data from each electrode is maximally independent of other electrodes and identifies artifacts or signals from non-cortical sources (e.g., muscle activity, eye-blink artifacts, pulse, etc.). Artifacts identified through ICA were removed using the multiple artifact rejection algorithm (MARA) plug-in (Winkler et al., 2011, Winkler et al., 2014). Since the data was collected from a 64-electrode cap, the data was re-referenced using the average across the entire scalp. Continuous data was epoched from − 500 msec before to 1000 msec after each content word was presented. Each EEG epoch in response to a content word constitutes a single trial. Epoched trials were rejected if peak to peak activity, within a moving window, or step-like activity, was greater than 100 uV. On average, 91.1% of trials were retained (656.08 trials out of 720; *SD* = 123.39), and an average of 7.88 components were removed (*SD* = 3.77).

### 2.6 Analysis of event-related potentials (ERPs)

Event-related potentials (ERPs) were calculated using the ERPlab toolbox within Matlab (Lopez-Calderon and Luck, 2014; code is publicly available^1^). For each trial and electrode, the mean amplitude of the pre-stimulus interval (−500 to 0 ms) was subtracted from each time point in the post-stimulus interval to correct for baseline differences. Single trials were then averaged together to obtain a stable waveform ERP for each electrode for every subject. Plots of ERP amplitude across all trials at each ROI are included in Supplementary Fig. 1.

### 2.7 Analysis of event-related spectral perturbations (ERSPs)

Event-related spectral perturbations (ERSPs) were calculated using code adopted from Fieldtrip (Oostenveld et al., 2011; code publicly available^2^). We applied a single taper Hanning window with a length of 500 ms across 25 ms steps of the 1.5 s epochs. The resulting transformation resulted in ERSP data across a frequency range of 3–30 Hz (1 Hz steps). A pre-content word (−500 to 0 ms) baseline was subtracted to examine neural activity changes in response to content word presentation. The baseline correction procedure was performed on a single-trial basis, resulting in 4D data structures containing Trial x Channel x Frequency x Time information for each subject. Plots of theta activation across all trials at each ROI are included in Supplementary Fig. 1.

### 2.8 Extraction of ERP and ERSP Data

**Fig. 1:**
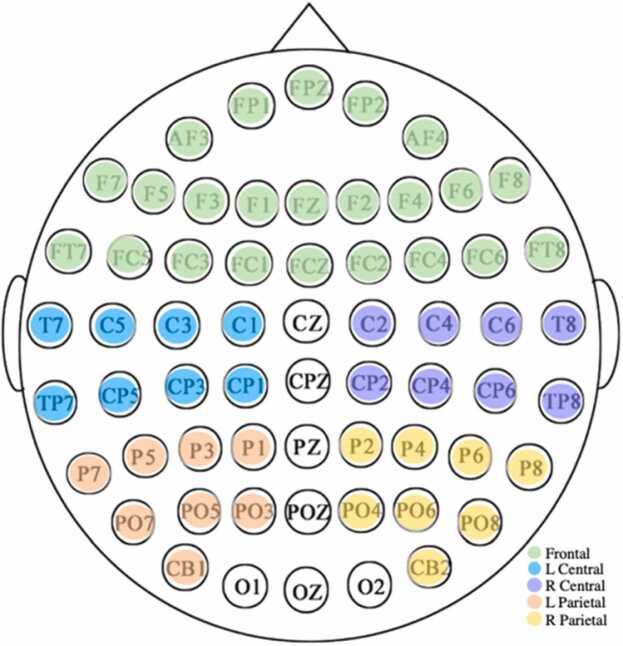
Regions of Interest (ROIs) utilized in the current study.

ERP and ERSP data were extracted from 5 regions of interest (ROIs; Fig. 1): frontal, left central, right central, left parietal and right parietal. ERP and ERSP data were averaged across all electrodes within each ROI. By averaging across the theta frequency (4–8 Hz), we were able to extract the mean amplitude of the ERSP within each ROI from 0 to 1000 ms in 25 ms steps (a total of 40 time points). ERP instantaneous amplitude was extracted from each ROI at the same time points from 0 to 1000 ms in 25 ms steps. Analysis scripts are publicly available.^3^

### 2.9 Statistical analysis

In the current study, we focus on the retrieval of individual word meanings while reading a sentence presented word-by-word, by examining individual differences in the N400 and theta response elicited by content words. To accomplish this goal, we utilized single-trial multilevel modeling to model individual differences related to age and language ability separately on measures of neural activity (N400, theta). All regression modeling was performed in R (RStudio Team, 2018) using the lme4 package (Bates et al., 2015).^4^ We implemented two linear mixed effects models (LMER). The first model included single-trial measurements of the ERP waveform between 300 and 500 ms, and the second included single-trial measurements of ERSP data corresponding to the theta frequency (4–8 Hz) between 300 and 500 ms. EEG data was averaged across electrodes located within each of the 5 ROIs cluster (see Fig. 1 for ROI configuration details) at each time point. Our analyses focus on these ROIs to reduce the computational load associated with our regression analysis. Fixed effects across both models included maternal education level (mean centered) and language experience, as well as interaction terms between the following variables and each ROI: chronological age (mean centered), PPVT standard score (z-normed), GORT comprehension scaled score (z-normed), and NWR percent consonants correct (z-normed). To evaluate the association between the N400 and theta, and to isolate the influence of age and language ability, the ERP model included fixed effects for the interaction between ROI and theta amplitude, and the ERSP model included fixed effects for the interaction between ROI and N400 amplitude. All models included random intercepts to reduce estimation error associated with subject-level variance not related to variables of interest (Baayen et al., 2008).

## 3 Results

### 3.1 N400 ERP LMER

**Fig. 2:**
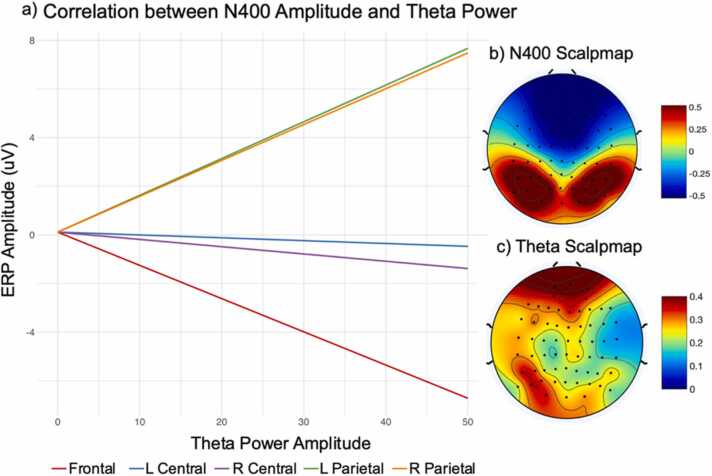
The direction of the correlation between ERP amplitude and theta power shifts across ROIs. a) At frontal electrodes increasing theta power was associated with decreasing N400 amplitude (red line); however, N400 amplitude increased with increasing theta power at more parietal sites (orange and green lines). The association between theta amplitude and the N400 was significant at all ROIs except the left central region (blue line). Plots represent predicted values, controlling for all other variables included in LMER model. b) Scalpmap of N400 ERP activation between 300 and 500 msec after word onset. Red indicates increases in activation and blue denotes decreases in activation, relative to baseline. c) Scalpmap of theta ERSP activation between 300 and 500 msec after word onset. Theta power increases were widespread, with less of an increase in activation represented by blue, and greater increases in theta represented by red.

A LMER model with ERP amplitude between 300 and 500 ms was used to examine the relationship between the N400 and theta power, and whether individual differences related to age and language abilities uniquely influence the N400 during word retrieval. The results from this LMER model revealed significant interaction terms between theta amplitude and ROI across all regions except left central electrodes. Theta amplitude between 300 and 500 ms was negatively associated with N400 amplitude at frontal and right central electrode sites; however, this association was positive at bilateral posterior regions (see Fig. 2).

**Fig. 3:**
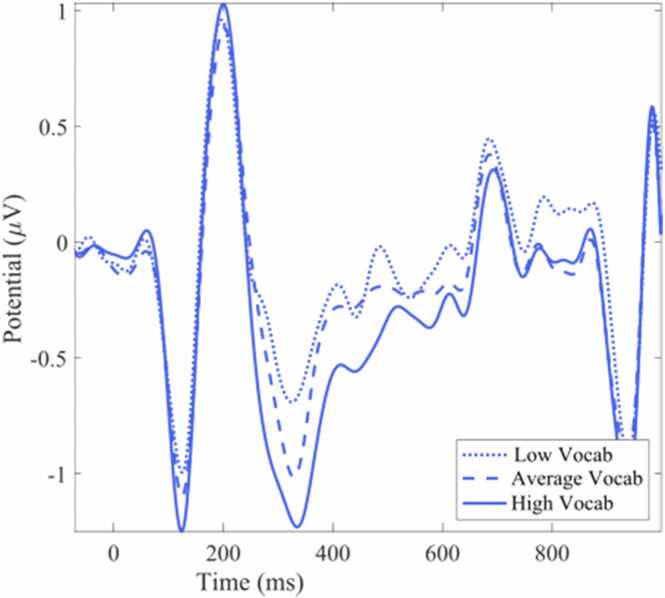
ERP N400 waveform based on PPVT vocabulary scores averaged over left central ROI. Increasing vocabulary knowledge was associated with greater negativity between 300 and 500 ms. Vocabulary score was included as a continuous variable in all analyses. For plotting purposes, individuals were grouped based on the median of our sample. PPVT standard scores of under 96 (25th percentile) were classified as Low Vocab, scores above 120 (75th percentile) were classified as High Vocab, and all others as Average Vocab.

Age and N400 amplitude were negatively associated in frontal and left parietal regions, suggesting that N400 amplitude decreases as a function of increasing age. Vocabulary knowledge, as measured by the PPVT, was negatively associated with N400 amplitude at left central electrodes, suggesting that N400 amplitude decreases as a function of increasing vocabulary knowledge (Fig. 3). The full output of the ERP LMER model can be found in Table 3.

**Table 3:** Complete output of LMER model with N400 ERP Amplitude as dependent variable.

	N400 ERP Amplitude		
*Predictors*	*Estimates*	*CI*	*p*
(Intercept)	0.13	0.07 – 0.19	**< 0.001**^a^
Maternal Education	0.00	-0.03 – 0.03	1.000
Language Experience	-0.02	-0.10 – 0.05	0.541
Theta * Frontal ROI	-0.14	-0.15 – − 0.12	**< 0.001**^a^
Theta * L Central ROI	-0.01	-0.03 – 0.01	0.207
Theta * L Parietal ROI	0.15	0.14 – 0.16	**< 0.001**^a^
Theta * R Central ROI	-0.03	-0.05 – − 0.01	**0.001**^b^
Theta * R Parietal ROI	0.15	0.13 – 0.16	**< 0.001**^a^
Age * Frontal ROI	-0.05	-0.09 – − 0.02	**0.002**^b^
Age * L Central ROI	0.02	-0.02 – 0.05	0.364
Age * L Parietal ROI	0.05	0.02 – 0.09	**0.003**^b^
Age * R Central ROI	-0.03	-0.06 – 0.00	0.086
Age * R Parietal ROI	0.02	-0.01 – 0.06	0.182
Vocabulary * Frontal ROI	-0.05	-0.16 – 0.07	0.434
Vocabulary * L Central ROI	-0.13	-0.24 – − 0.01	**0.032**^c^
Vocabulary * L Parietal ROI	-0.02	-0.13 – 0.10	0.779
Vocabulary * R Central ROI	-0.06	-0.18 – 0.05	0.278
Vocabulary * R Parietal ROI	0.00	-0.11 – 0.12	0.980
Reading Comp. * Frontal ROI	0.02	-0.09 – 0.13	0.695
Reading Comp. * L Central ROI	0.10	-0.01 – 0.20	0.088
Reading Comp. * L Parietal ROI	0.07	-0.04 – 0.18	0.195
Reading Comp. * R Central ROI	-0.01	-0.12 – 0.10	0.804
Reading Comp. * R Parietal ROI	-0.00	-0.11 – 0.11	0.954
Phon. Memory * Frontal ROI	0.04	-0.04 – 0.12	0.385
Phon. Memory * L Central ROI	-0.00	-0.08 – 0.08	0.965
Phon. Memory * L Parietal ROI	-0.02	-0.10 – 0.06	0.582
Phon. Memory * R Central ROI	-0.01	-0.09 – 0.07	0.758
Phon. Memory * R Parietal ROI	-0.02	-0.10 – 0.06	0.606
**Random Effects**			
σ^2^	0.30		
Observations	1085		
Marginal R^2^ / Conditional R^2^	0.605 / NA		

a *p* < 0.001.

b *p* < 0.01.

c *p* < 0.05.

### 3.2 Theta ERSP LMER

**Fig. 4:**
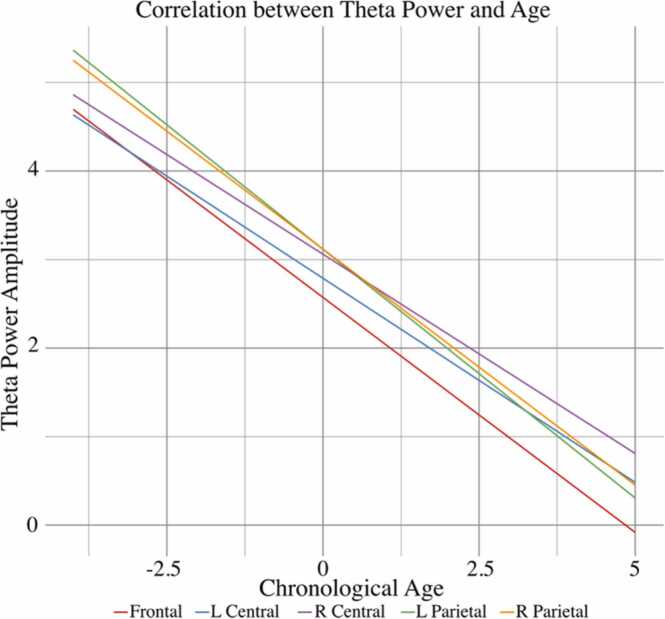
Theta amplitude across the scalp decreases as a function of age. Plots represent predicted values, controlling for all other variables included in LMER model.

A LMER model with theta amplitude between 300 and 500 ms was used to test the hypothesis that individual differences related to age and language abilities uniquely influence the theta response during word retrieval. Similar to the ERP Model, the results from this LMER model revealed significant interaction terms between N400 ERP amplitude and ROI across all regions except left central electrodes. Theta amplitude was negatively associated with N400 ERP amplitude at frontal electrodes but shifted towards a positive relationship at right central and bilateral posterior electrodes. Additionally, age was negatively associated with theta amplitude across all ROIs, indicating that theta amplitude during word retrieval decreases as a function of increasing age (see Fig. 4). There was no significant relationship between vocabulary and theta amplitude. The full output of the ERSP LMER model can be found in Table 4.

**Table 4:** Complete output of LMER model with Theta Power Amplitude as dependent variable.

	Theta Power Amplitude		
*Predictors*	*Estimates*	*CI*	*p*
(Intercept)	3.00	2.22 – 3.78	**< 0.001**^a^
Maternal Education	-0.19	-0.57 – 0.18	0.310
Language Experience	-0.38	-1.50 – 0.75	0.515
N400 * Frontal ROI	-1.09	-1.38 – − 0.79	**< 0.001**^a^
N400 * L Central ROI	-0.08	-0.56 – 0.41	0.763
N400 * L Parietal ROI	1.46	1.27 – 1.66	**< 0.001**^a^
N400 * R Central ROI	1.19	0.59 – 1.79	**< 0.001**^a^
N400 * R Parietal ROI	1.46	1.26 – 1.66	**< 0.001**^a^
Age * Frontal ROI	-0.54	-0.77 – − 0.31	**< 0.001**^a^
Age * L Central ROI	-0.47	-0.70 – − 0.24	**< 0.001**^a^
Age * L Parietal ROI	-0.57	-0.80 – − 0.34	**< 0.001**^a^
Age * R Central ROI	-0.46	-0.69 – − 0.22	**< 0.001**^a^
Age * R Parietal ROI	-0.54	-0.77 – − 0.31	**< 0.001**^a^
Vocabulary * Frontal ROI	0.05	-0.79 – 0.90	0.899
Vocabulary * L Central ROI	0.23	-0.62 – 1.07	0.600
Vocabulary * L Parietal ROI	0.32	-0.52 – 1.16	0.457
Vocabulary * R Central ROI	0.54	-0.31 – 1.38	0.213
Vocabulary * R Parietal ROI	0.55	-0.30 – 1.39	0.204
Reading Comp. * Frontal ROI	0.29	-0.47 – 1.05	0.451
Reading Comp. * L Central ROI	0.20	-0.56 – 0.96	0.603
Reading Comp. * L Parietal ROI	0.07	-0.69 – 0.83	0.853
Reading Comp. * R Central ROI	0.12	-0.64 – 0.88	0.757
Reading Comp. * R Parietal ROI	0.06	-0.70 – 0.81	0.886
Phon. Memory * Frontal ROI	0.29	-0.27 – 0.85	0.316
Phon. Memory * L Central ROI	0.23	-0.33 – 0.79	0.429
Phon. Memory * L Parietal ROI	0.12	-0.44 – 0.68	0.674
Phon. Memory * R Central ROI	0.18	-0.38 – 0.74	0.525
Phon. Memory * R Parietal ROI	0.16	-0.40 – 0.72	0.574
**Random Effects**			
σ^2^	2.29		
Observations	1085		
Marginal R^2^ / Conditional R^2^	0.170 / 0.869		

* * *p* < 0.01, * *p* < 0.05.

a *p* < 0.001.

## 4 Discussion

This study is the first to address the relationship between the N400 and theta response during semantic retrieval of individual words through middle childhood and adolescence, and specify unique, developmental predictors of each. Our findings indicate a strong relationship between the N400 and theta responses in school-age children. This relationship varies over the surface of scalp, with the two neural responses being negatively correlated over frontal areas, but positively correlated over posterior areas. Importantly though, this association is influenced by other developmental factors, such as age and vocabulary knowledge. These findings provide essential, foundational information about the development of the neural correlates underlying semantic retrieval and individual characteristics which may influence each component. We speculate that the N400 and theta response provide unique, important information about how the developing brain processes language.

In line with previous work, we revealed that the N400 and theta are associated across the scalp (Davidson and Indefrey, 2007); however, our findings indicate that this association is different between frontal and parietal ROIs. Although many studies implementing semantic tasks have highlighted the similarity between the N400 and theta response, few studies have directly compared these responses (for review, see Schneider and Maguire, 2018). In adults, an inverse relationship between the N400 and theta is typically reported (Davidson and Indefrey, 2007, Schneider et al., 2016, Schneider and Maguire, 2018a); however, the current findings indicate a more complex relationship emerges across different ROIs in children. Our findings point to an inverse relationship between the N400 and theta in children across frontal electrodes, but this relationship becomes more positive as it shifts parietally. While the location of the N400 varies based on task demands, in adults, the source of the N400 is believed to be in the posterior half of the left superior temporal gyrus, and spreads throughout bilateral tempo parietal cortices (Kutas and Federmeier, 2011). Based on this knowledge, a more negative N400 waveform should be expected at parietal sites. Meanwhile, widespread theta increases have also been reported in adult studies of semantic processing (Maguire et al., 2022, Schneider et al., 2016). Therefore, this inverse relationship at parietal sites is likely due to a more negative N400 waveform and theta power increase in response to higher processing demands placed on retrieval of semantic information (Bastiaansen et al., 2008a, Bastiaansen et al., 2002, Schneider et al., 2016).

Building on this, we speculate that the positive correlation at frontal sites during semantic retrieval of content words from sentences observed here is attributed to a more positive N400 response in frontal regions and widespread theta increases. Therefore, theta may be sensitive to the widespread recruitment of semantic processes across the scalp, while the N400 is gauging complementary processes limited to the parietal cortex. These findings have important practical applications for EEG studies of semantic development because they suggest variability across different ROIs may heavily influence the relationship between the N400 and theta. It is also interesting to note that the relationship between the N400 and theta was lacking over left central electrodes. This region is particularly important for semantic processing (for review, see Binder and Desai, 2011; Friederici, 2011), as a meta-analysis of 120 neuroimaging studies revealed the middle temporal gyrus and posterior inferior parietal lobe were shown to play a role in semantic integration and the left inferior frontal gyrus played a critical role in semantic processing. While EEG lacks the spatial localization capabilities of fMRI, the neural response in this left central ROI appears to be most influenced by developmental factors, such as language abilities and age.

The N400 has been proposed as gauging individual differences in the breadth, depth and speed of semantic retrieval (Holcomb et al., 1992, Perfetti, 2007). Identifying the extent to which individuals vary in their N400 response to word meanings can inform us about implicit representations of language. Studies have shown the N400 is sensitive to individual differences in semantic processing in infants (Friedrich and Friederici, 2004) and adults (Landi and Perfetti, 2007); however, middle childhood is relatively understudied. In a study of 8–10-year-olds, Henderson et al. (2011) did not uncover an association between N400 amplitude and vocabulary; however, N400 amplitude was computed by averaging across the entire left hemisphere. In the current study, N400 amplitude at left-central electrodes varies based on vocabulary knowledge, although this association was weak in comparison to other associations in the current analysis. Given that this association was relatively weak in comparison, we carefully speculate that breadth, depth and speed of word retrieval in this region is sensitive to individual differences in vocabulary knowledge. One reason this relationship between vocabulary and N400 amplitude may not be as strong as in previous work is that EEG trials in the current study included a wide range of content words occurring throughout the course of a sentence. It may be that the relationship between the N400 and vocabulary varies based on word class, word concreteness or frequency, and predictability of each word within the sentence context. The fact that the relationship emerges despite wide variability in the content words themselves, speaks to the general stability of this effect. Furthermore, amplitude of the N400 during semantic retrieval varies as a function of age, decreasing at frontal and left parietal electrodes as a function of increasing age. These findings add to studies of the N400 in younger children (5–6-year-olds) showing a broadly distributed N400 in frontal, centroparietal and temporal channels that becomes increasingly localized to centroparietal channels with age, reflecting the fine-tuning of information-processing strategies across development (Byrne et al., 1999). Taken together, our findings suggest that the neural activation as measured by the N400 is moderately sensitive to vocabulary differences, above and beyond the influence of age and features specific to the word, at left-central electrodes.

In studies with adults, increases in theta are often observed in response to individual words in isolation, word pairs, and sentences (Bastiaansen et al., 2005, Bastiaansen et al., 2008b, Bastiaansen and Hagoort, 2015, Hald et al., 2006, Lam et al., 2016, Meyer, 2018, Schneider et al., 2016, Schneider et al., 2018, Schneider and Maguire, 2018b). While studies of theta responses in children during semantic retrieval are relatively sparse in comparison, evidence suggests that children exhibit increases in theta amplitude during individual word retrieval (Krause et al., 2000, Spironelli and Angrilli, 2010), while processing incongruent word pairs (Fernández et al., 2012), and when identifying incongruencies in sentences (Schneider and Maguire, 2018b, Spironelli and Angrilli, 2010). Importantly though, there is increasing evidence that the theta amplitude (Schneider and Maguire, 2018b) and topography (Maguire et al., 2022; Schneider et al., 2018b; Spironelli and Angrilli, 2010) vary as a function of increasing age. Specifically, Maguire et al. (2022) found that the theta response to known words within a sentence context a) localized to left-central regions and b) decreased in amplitude as children’s age increased from 8 to 15 years. The current study supports these findings, as maturation accounts for individual differences observed in the theta response, above and beyond the influence of language ability.

One significant strength of this study is that it included children from a diverse range of socioeconomic backgrounds, with a range of language abilities and language experiences. Importantly, socioeconomic status (SES) and language experience (bilingualism) were not significant predictors of individual differences in the N400 or theta during processing of content words. Thus, it appears individual language abilities and age are stronger predictors of differences in semantic retrieval than SES and bilingualism in the current sample. It should not be overlooked though that the current study used maternal education as a proxy for SES, therefore it is possible that other environmental differences linked to SES, such as differences in language input, may have an association with the development of semantic processing. Previous studies have demonstrated that the N400 is sensitive to differences in reading comprehension, but not phonological processing (Landi and Perfetti, 2007). While it is therefore not surprising that our measure of phonological working memory did not emerge as a significant predictor, it is interesting reading comprehension had no significant influence on N400 or theta response. We believe the lack of significant reading comprehension results may be attributed to the task, which included only easy, early acquired words. Therefore, the task may have been too simple for children ages 8–16 for their reading comprehension to be taxed. It is also possible that changes in resting state EEG may contribute to the differences in the N400 and theta uncovered in the current study (Campbell and Feinberg, 2009, Cellier et al., 2021, Perone et al., 2018). However, by baseline correcting the data we have tried to offset some of these potential differences, and as a result, feel confident that the N400 and theta changes reported here are driven primarily by brain maturation and changes in language abilities.

One limitation of the current study is that we did not account for differences among individual content words, such as word frequency, concreteness, word class, and semantic expectancy or relatedness within the sentence context. All of these factors are known to influence the N400 response to individual words. It is likely that each of these word-level factors may uniquely contribute to the theta and N400 responses and differ with age and vocabulary ability. This question is beyond the scope of the current study, which is primarily focused on the relationship between the N400 and theta to content word processing within an individual. However, future research should systematically investigate how characteristics of individual content words differentially influence the relationship between the N400 and theta oscillations. Another limitation relates to the fact that our ERSP analyses captured both induced and evoked theta responses. Evoked and induced oscillations differ in their phase-relationships to the stimulus: evoked oscillations are phase locked to the stimulus, whereas induced oscillations are not. Traditional ERPs capture evoked oscillations only, while ERSPs capture both induced and evoked oscillations. While we cannot definitively disentangle whether the N400 and induced theta response examined here are completely related, we believe examining both induced and evoked theta oscillations is highly informative for the field of developmental neuroscience, which largely examines theta oscillations containing both induced/evoked responses.

The study of neural oscillations is relatively new, especially in the field of developmental cognitive neuroscience. It is critical to establish neural oscillations in children to the decades of work in ERPs. Our findings accomplish this by demonstrating that while the N400 and theta may be similarly engaged during semantic retrieval, their association varies across the scalp, across the course of development, and as a function of vocabulary knowledge. These factors are not only important to consider in studies of typically developing children, but also when studying neurodiverse children or children with language disorders who may exhibit unique developmental and linguistic trajectories in their semantic processing skills.

## Funding

This work was supported by the National Science Foundation Developmental Sciences Division under Grant 1551770 (PIs: Maguire and Abel).

## Declaration of Competing Interest

The authors declare that they have no known competing financial interests or personal relationships that could have appeared to influence the work reported in this paper.

## Data Availability

Data and analysis scripts are publicly available at: https://github.com/juliagoolia28/manuscripts/tree/master/N400_Theta_Dev

## Footnotes

1 https://github.com/juliagoolia28/manuscripts/blob/master/N400_Theta_Dev/matlab_scripts/ft_preproc.m

2 https://github.com/juliagoolia28/manuscripts/blob/master/N400_Theta_Dev/matlab_scripts/ft_multivariate.m

3 https://github.com/juliagoolia28/manuscripts/blob/master/N400_Theta_Dev/matlab_scripts/data_extract_erp.m

4 https://github.com/juliagoolia28/manuscripts/blob/master/N400_Theta_Dev/r_scripts/erp_ersp_oc_analysis_ROIs.Rmd
