# Supplementary material for: Age and vocabulary knowledge differentially influence the N400 and theta responses during semantic retrieval
Source: Dev Cogn Neurosci. 2023 May 2;61:101251. doi: 10.1016/j.dcn.2023.101251 (PMC10311145; doi:10.1016/j.dcn.2023.101251)
Supplement: Supplementary file 1 — Supplementary material [file mmc1.docx]

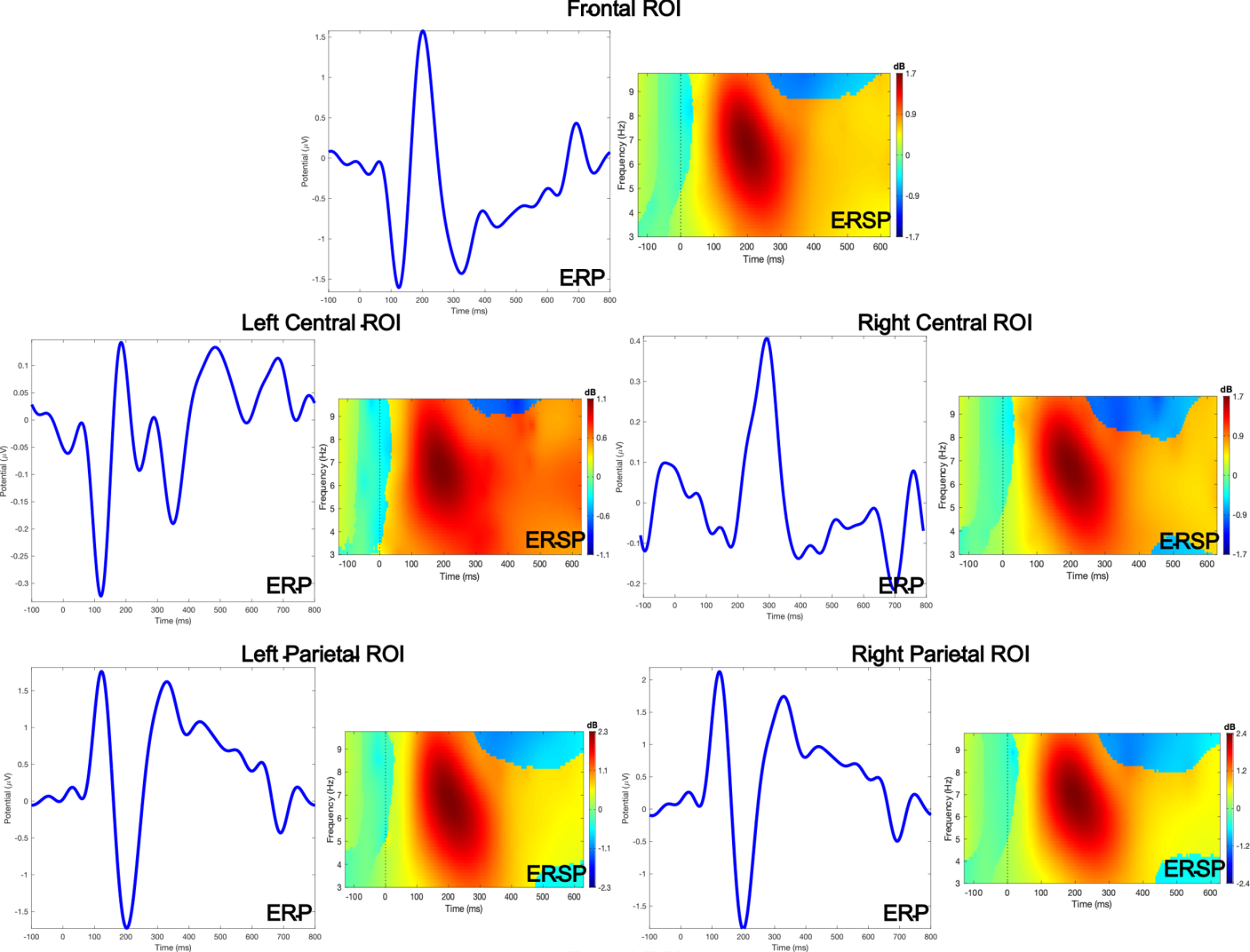


**Supplementary Figure 1**. Polarity of the N400 component and theta power at individual ROIs. Negative associations between the N400 component and theta power were reported at frontal and central ROIs, while positive associations between the two were present at parietal ROIs.
